# Is it a Match? Yawn Contagion and Smile Mimicry in Toddlers

**DOI:** 10.1007/s12110-025-09488-8

**Published:** 2025-03-13

**Authors:** Ivan Norscia, Marta Caselli, Chiara Scianna, Sara Morone, Martina Brescini, Giada Cordoni

**Affiliations:** Department of Life Sciences and Systems Biology, University of Torino (DBIOS), Via Accademia Albertina 13, 20123 Turin, Italy

**Keywords:** Motor resonance, Facial mimicry, Behavioral matching, Emotional contagion, Empathy, Emotion ontogeny

## Abstract

Automatic behavioral matching includes Rapid Facial Mimicry (RFM) and Yawn Contagion (YC) that occur when the facial expression of an individual acts as a ‘mirror social releaser’ and induces the same facial expression in the observer (within 1 s for RFM, and minutes for YC). Motor replication has been linked to coordination and emotional contagion, a basic form of empathy. We investigated the presence and modulating factors of Rapid Smile Mimicry (RSM) and YC in infants/toddlers from 10 to 36 months at the nursery ‘Melis’ (Turin, Italy). In February-May 2022, we gathered audio and/or video of all occurrences data on affiliative behaviors, smiling during play, and yawning during everyday activities. Both RSM and YC were present, as toddlers were most likely to smile (within 1 s) or yawn (within three-min) after perceiving a smile/yawn from another toddler. Sex, age, and parents’ country of origin did not influence RSM and YC occurrence, probably because gonadal maturation was long to come, the age range was skewed towards the early developmental phase, and toddlers had been in the same social group for months. RSM and YC showed social modulation, thus possibly implying more than just motor resonance. Both phenomena were inversely related to affiliation levels (a social bond proxy). Because literature reports that in adults RSM and YC may increase with familiarity, our reversed result suggests that in certain toddler cohorts the same phenomena may help increase socio-emotional coordination and that the function of motoric resonance may be experience- and context-dependent.

## Introduction

Behavioral matching is a form of motor replication that takes place when an observed behavior induces the observer to repeat it (Gallese, 2004; Schütz-Bosbach & Prinz, 2015; Zentall, 2003). Behavioral matching can occur at different cognitive levels ranging from automatically mirroring others’ motor patterns to ‘copying’ phenomena that require high-level cognitive appraisal (e.g., true imitation, emulation; Zentall, 2012). Depending on cognitive complexity and self-other distinction abilities, behavioral matching can have different social repercussions, such as emotional transfer, social learning facilitation, and promotion of social relations by fostering affiliation and cooperation (Berthier & Semple, 2018; Canteloup et al., 2020; de Waal & Preston, 2017; Panksepp & Panksepp, 2013; Paukner et al., 2009).

The most basic level of behavioral matching is automatic motor replication, a broad umbrella concept that includes motor mimicry and behavioral contagion (Palagi et al., 2020), because both can be mediated by the Perception–Action Model (PAM) and the Mirror Neuron System (MNS; de Waal & Preston, 2017; Rizzolatti & Caruana, 2017). According to PAM and MNS, for both phenomena the observation of the motor pattern activates in the observer the same motor neurons as in the individual performing such a pattern, with the focus being on the goal more than on the action (MNS; Rizzolatti & Caruana, 2017; Rizzolatti & Fabbri-Destro, 2010; Schütz-Bosbach & Prinz, 2015) and with the observer's response being shaped by their own experience (PAM; Preston & de Waal, 2017). However, motor mimicry differs from behavioral contagion because the former involves the observer repeating others’ motor patterns within a short time window (within 1 s or a few seconds), whereas the latter involves the observer replicating the behavior but not necessarily the exact actions of others over longer time windows (ranging from less than 1 s to several minutes; Palagi et al., 2020; Prochazkova & Kret, 2017; Wheeler, 1966; Zentall, 2003). Depending on the behavior being considered, behavioral contagion can also involve a physiological component, which may account for the delayed response often observed in contagion compared to pure mimicry (Prochazkova & Kret, 2017). In both cases, the behavioral pattern performed by an individual and perceived by another individual (via vision or other sensory cues) works as a ‘social releaser’ (sensu Tinbergen, 1951). According to Konrad (1935), social relations are dependent on a wide array of stimuli emitted by one individual (the “actor”) that release responses in another individual (the “reactor”). Based on this, Tinbergen (1948, 1951) showed that innate social responses are dependent on the display of releasers and better defined the social dimensions of releasers. In the case of automatic motor replication, others’ motor patterns not only act as social releasers (sensu Tinbergen, 1951) but generate in the perceiver a specific ‘mirror response’. Motor mimicry and contagion can occur with different ‘mirror social releasers’, as we may define them, and translate the motor pattern from the individual to the social level.

A form of motor mimicry is Rapid Facial Mimicry (RFM), where the facial expression displayed by an individual (e.g., smile in Rapid Smile Mimicry, RSM) induces the observer to reproduce the same expression within 1 s (de Waal & Preston, 2017; Iacoboni, 2009; Palagi et al., 2020; Schütz-Bosbach & Prinz, 2015; Zentall, 2003). A form of behavioral contagion is yawn contagion, which occurs when a perceived yawn triggers a yawning response in the perceiver (Provine, 1989a, 1989b). Such a response can be within 1 s, a few seconds, or even minutes from the exposure to the yawning stimulus (Palagi et al., 2020; Provine, 2005). The relevance of investigating facial mimicry and yawn contagion is that they may be related to emotional contagion, an automatic and implicit form of empathy (Preston & de Waal, 2002, 2017). It has been hypothesized that automatic motor replication via facial mimicry and yawn contagion may have functioned as an exaptation for emotional contagion (Hess & Fischer, 2013; Palagi et al., 2020). By observing the facial expression of another person, the observer may activate not only the motor neurons connected with the expression but also shared representations of the emotion that such expression conveys (de Waal & Preston, 2017).

In infants and toddlers, smiling is considered the expression of positive emotions (Messinger et al., 2008). Smiles can involve a wide combination of different facial unit movements, but four main types may be recognized based on mouth opening and eye-constriction: simple/basic smile (mouth closed/no eye constriction), play smile (mouth open, no eye constriction), Duchenne smile (mouth closed/eye constriction), and duplay smile (mouth open/eye constriction; Fogel et al., 2006; Messinger et al., 2008). Although play smiles are the most common during play (as the name indicates), the other types of smiles can be variably present during this behavior (Dickson et al., 1997; Fogel et al., 2006). Hence, social play is one of the most suitable behaviors to consider when investigating facial mimicry, as play sessions are visually punctuated by smiles.

Rapid facial mimicry of playful facial expressions may be biologically ancient as it has been observed in humans (Seibt et al., 2015), other hominids (orangutans—*Pongo pygmaeus*: Davila-Ross et al., 2008; chimpanzees – *Pan troglodytes*—and gorillas – *Gorilla gorilla*: Palagi et al., 2019; bonobos – *Pan paniscus*: Bertini et al., 2022), and other animal species (spanning monkeys, rodents and carnivores; Palagi et al., 2020), where they are termed ‘play faces’ or ‘relaxed-open mouth’. This observation suggests deep mammalian roots of the phenomenon. Automatic facial expression replication (not necessarily related to true imitation), including smile, is observed since the first phases of life in human infants (but also non-human primates; Ferrari et al., 2006; Jones, 2009; Wörmann et al., 2014). Automatic facial mimicry in humans appears to be present as early as 5 months of age, when multimodal emotional stimuli are elaborated (Isomura & Nakano, 2016). Smile mimicry has been described in adult humans as well (e.g., Mui et al., 2018; Seibt et al., 2015). Individual and social factors may influence facial mimicry, such as age, sex, group membership, and social bond (Seibt et al., 2015). Facial emotion recognition skills change with age (e.g., in relation to sensitivity to different emotions or increase of finely tuned discrimination abilities) and as early as in the first two years of life cognitive and mimicking abilities of toddlers increase in variety, latency, and complexity (Grossman et al., 2007; Hühnel et al., 2014; Jones, 2009; Lawrence et al., 2015). Hence, this can potentially affect the extent to which facial mimicry is expressed—as age increases—during naturally occurring social interactions. Moreover, human females show possibly more precise and/or effective processing of emotional facial expressions and increased facial mimicry than men, as a result of possible inter-sex differences in the smile facial mimicry neural network (Dimberg & Lundquist, 1990; Hall, 1978; Hall & Matsumoto, 2004; Hoffmann et al., 2010; Korb et al., 2015). Additionally, group membership—including country of origin and ethnicity—can influence the amount of mimicry response to others’ facial expressions (also according to type, e.g., happy *vs* angry) in young children and adults (de Klerk et al., 2019; Rauchbauer et al., 2016; Seibt et al., 2015). Finally, facial mimicry can be influenced by different social setting variables, including the social relationship between interactants. Although few studies have investigated this aspect, facial mimicry—including smile mimicry—may increase when the social bond is tight (Fischer et al., 2012; Häfner & Ijzerman, 2011).

Although understudied, a modulation of individual factors on the rapid facial mimicry of the play face has been observed in non-human primates, including hominid species (e.g., sex combination of interactants, lowland gorillas: Bresciani et al., 2022; age, orangutans: Davila-Ross et al., 2008). Moreover, a positive influence of the social relationship on play face facial mimicry rates has been found in non-human mammals, such as dogs (Palagi et al., 2015). Thus, the modulation of rapid facial mimicry of smile may be rooted in mammalian evolution and vary depending on species biology.

Contrary to smile, yawning can be associated with physiological transitions (e.g., from sleep to wake) and also negative internal states, such as boredom, tiredness, or mild stress (Guggisberg et al., 2010; Thompson, 2014; Zilli et al., 2007). The yawning-like motor pattern is likely a plesiomorphic display as it has been described in a wide range of vertebrates, including non-human primates (Anderson, 2020; Baenninger, 1997). Besides humans (Provine, 1989a, 1989b), yawn contagion has been reported in a variety of animal species (mammals, for review: Palagi et al., 2020; bird: *Melopsittacus undulatus*; Gallup et al., 2015), including hominids (e.g., orangutans: van Berlo et al., 2020; chimpanzees: Anderson et al., 2004; bonobo: Demuru & Palagi, 2012; but not gorillas: Palagi et al., 2019). Thus, yawn contagion, as facial mimicry, might stem from ancient evolutionary foundations, although the variation in the reported expression of the phenomenon may relate to species-specific social features more than to phylogeny, and/or to sample limitations (Palagi et al., 2020; van Berlo et al., 2020).

As facial mimicry, in humans also yawn contagion can be affected by individual and social factors. The influence of sex on yawn contagion rates is still under debate as it was found in certain cohorts of adult humans (with females showing more contagion, e.g., Chan & Tseng, 2017; Norscia et al., 2016) but not in others (e.g., Bartholomew & Cirulli, 2014; Norscia & Palagi, 2011), with cultural features possibly enhancing variability (Palagi et al., 2020). No sex effect on yawn contagion was found in children from 2.5 to 5.5 years old (Cordoni et al., 2021). Contrary to sex, age is a critical variable influencing yawn contagion. As a matter of fact, yawn contagion increases during ontogenetic development, with children by the age of 10–11 years showing contagion levels similar to adults (Anderson & Meno, 2003). Previous reports that used yawn video stimuli found that infants and toddlers (6 to 34 months old) did not respond to their mothers’ yawns from 6 to 34 months old (Miller and Anderson, 2011) or others’ yawns up to 5 years old (Anderson & Meno, 2003). Helt et al. (2010)—by exposing children to real yawns emitted by a live, adult subject—found that yawn contagion increased with age from 1 to 5–6 years old, being virtually absent between 1 and 2 years of age (0–5% of toddlers showing a yawn response at least in one trial) and still infrequent by the age of 3 (10% of toddlers responding). An increase to 35–40% was observed in children from 4 to 5–6 years old. However, no analysis is available on the presence or absence of the phenomenon at the group level for different age classes. A study investigating yawn contagion in a naturalistic social setting, during children’s everyday activities in a nursery, found that yawn contagion was already present at 2.5 years of age (Cordoni et al., 2021). The study did not investigate the possible presence of yawn contagion before that age as younger toddlers were not present in the sample. Finally, literature on adults shows that country of origin differences may not affect yawn contagion whereas stronger social relationships between individuals increase yawn contagion probability in certain human cohorts (Norscia & Palagi, 2011; Norscia et al., 2020, 2021). Although this aspect is not known in children, such an effect cannot be excluded during the first phases of ontogeny, as the intersubjective ability to differently respond to others’ emotional expressions depending on familiarity appear early in infant development (Walker-Andrews et al., 2011).

The effect of individual (i.e., sex and age) and social factors (i.e., group membership and/or familiarity) in modulating the expression of yawn contagion may also be biologically rooted in human evolutionary history, as they can variably affect yawn contagion in non-human animals, including non-human hominins (chimpanzees and bonobos; e.g. Campbell & de Waal, 2011; Demuru & Palagi, 2012; Madsen et al., 2013; Massen et al., 2012; Norscia et al., 2022). In particular, social modulation may indicate that yawn contagion is not merely a phenomenon of motoric resonance (in which case it would be equally distributed across dyads) but that it may underlie an emotional transfer component (Norscia et al., 2021; Palagi et al., 2020; but see: Massen & Gallup, 2017).

In this study, we report for the first time on the presence and modulating factors of rapid facial mimicry and yawn contagion, as a possible proxy of emotional contagion, in early and late toddlers (from 10- to 36-month-old), mostly below 2.5 years of age. Based on the above framework, we put forth the following predictions.

### Prediction 1—Facial Mimicry of Smile

Facial mimicry—including smile mimicry—appears very early in the ontogeny of humans as an automatic process (de Waal & Preston, 2017; Ferrari et al., 2013; Isomura & Nakano, 2016). Hence, we expected to find Rapid Smile Mimicry (RSM) in early and late toddlers (Prediction 1a). Because in the initial two years of life, the cognitive and replication abilities of toddlers expand in typology, delay, and complexity (Grossman et al., 2007; Hühnel et al., 2014; Jones, 2009; Lawrence et al., 2015), we expected RSM to increase with age (Prediction 1b). Although women can show increased facial mimicry than men and possess differences in the smile mimicry neural network (Dimberg & Lundquist, 1990; Hoffmann et al., 2010; Korb et al., 2015), we expected sex not to influence RSM in toddlers (Prediction 1c), as they are well below the hormonal change that characterizes pubertal onset and gonadal maturation (Abreu & Kauser, 2016). Finally, because belonging to the same group (including nationality) and tighter social relationships may increase mimicry responses (Fischer et al., 2012; Häfner & Ijzerman, 2011; de Klerk et al., 2019; Rauchbauer et al., 2016; Seibt et al., 2015), we expected RSM to be highest when toddlers shared a common geographical origin (Prediction 1d) or a close social relationship (Prediction 1e).

### Prediction 2—Yawn Contagion

Yawn contagion is considered an automatic phenomenon (de Waal & Preston, 2017; Prochazkova & Kret, 2017). Moreover, yawning responses to others’ yawns may start emerging from one to two years (Helt et al., 2010), and in naturally occurring interactions yawn contagion has been anticipated to 2.5 years of age (lower age limit of the sample; Cordoni et al., 2021). Hence, we hypothesized yawn contagion to be present starting in early toddlerhood (Prediction 2a). However, yawn contagion was reported to increase from two to three years of age, possibly remaining stable from 2.5 years onwards in pre-school years, with a further increase in school years (Cordoni et al., 2021; Helt et al., 2010). Hence, we expected yawn contagion to increase with age in our toddler cohort (Prediction 2b). Based on previous reports that did not find any effect of sex on yawn contagion in children (Cordoni et al., 2021) and no effect of country of origin on adult yawn contagion (Norscia & Palagi, 2011), we expected no effect of either toddler sex (Prediction 2c) or parents’ country of origin (Prediction 2d) on yawn contagion. Because the influence of familiarity on the responses to others’ emotional expression seems to emerge early in the development and stronger social relationships increase yawn contagion in adults (Norscia & Palagi, 2011; Walker-Andrews et al., 2011), we expected the same trend to be present in toddlers (Prediction 2e).

## Material And Methods

### Study Site and Groups

We carried out ethological observations from February to May 2022 at the nursery “Armando Melis” (Turin, Italy) on toddlers from two classrooms. Classroom “Pebbles” at the beginning of the study was composed of two infants (one male and one female, 10 and 12 months old respectively) and six early toddlers (five males and three females, from 13 to 14 months old). Classroom “Lavender” was composed of eight early toddlers (five males and three females, from 16 to 24 months old) and ten late toddlers (four males and six females, from 25 to 36 months-old). The two infants became toddlers in the first two months from the study start and three of the ten late toddlers became older than 36 during the study period (technically qualifying as ‘pre-schoolers’). Hence, our children were toddlers for most of the study period and will therefore be referred to as early and late toddlers from now on. The toddlers had been together for at least 5 months (as nursery frequentation started in September 2020).

The toddlers of each classroom could access a dedicated room for meals, free play and activities, a common ‘water’ room for activities with water, and a common garden which the toddlers from each classroom could access in turns. All the toddlers were born in Italy. The toddlers from classroom “Pebbles” had parents from Italy (five), Ecuador (one), Nigeria (one), Egypt/Morocco (one), Ivory Coast/Italy (one), Morocco/Italy (one). The toddlers from classroom “Lavender” had parents from Italy (nine), Romania (two), Peru (two), Nigeria (one), Nigeria/Benin (one), Albania/Italy (two), Romania/Italy (one). As per the information willingly and voluntarily provided by parents, no toddler had been certified for disabilities or disorders.

The typical day in the nursery was organized as follows: i) entrance from 8:30 to 9:30 h; ii) breakfast and activities guided by educators from 9:30 to 10:00 h; iii) free play and interactions from 10:00 to 11:00 h; iii) lunch from 11:00 to 12:00 h; iv) free play and interactions from 12:00 to 13:00 h; v) nap from 13:00—15:00 h; vi) snack, free play and interactions from 15:00 to 15:30 h; v) exit from 15:30 to 16:30 h.

### Data Collection and Operational Definitions

Behavioral data were audio and/or video recorded by two observers (S.M. and C.S.) three days per week spanning morning (08:00 am) and afternoon (03:30–04:00 pm) including lunch time. We gathered data via the all occurrences sampling method (Altmann, 1974) on social play, affiliative interactions, and yawning events. Social play and affiliation behavioral items are described in Table 1. In particular, videos on play interactions were recorded using a full HD camera (ZOOM-Q8 and Panasonic HC-V380) and subsequently analyzed frame-by-frame and/or slow-motion via freeware Avidemux 2.7.0. Before commencing systematic data collection, S.M and C.S. were trained for 25 h in behavioral pattern identification, sampling method utilization and data entry and sort out by M.C., G.C. and I.N. Training in behavioral pattern identification ended when Cohen's K value of 0.80 was reached (strong agreement; sensu McHugh, 2012).

**Table 1:** Ethogram of the play and affiliative behavioral patterns (not including play) used in this study (adapted from Piaget, 1964; Smith, 2009; Stone, 2007)

BEHAVIORAL PATTERN	DESCRIPTION
AFFILIATIVE PATTERNS	
Body contact	The child moves/sits/stands/lays down with a part of their body in contact with a part of the companion's body
Caress	The child uses their hands (usually the palm) to stroke gently a part of the companion's body
Embrace	The child holds the companion tightly with one or both arms
Kiss	The child touches with their closed lips a part of the companion 's body (usually the cheek)
Food/Object sharing	The child offers a portion of their food or an object (e.g., a toy) to the companion
PLAY PATTERNS	
Biting	The child closes gently their mouth on a part of the companion's body
Body wagging	Two (or more) children are in a four-legged position and move by swaying their buttocks from side to side as dog tail wagging
Bumper cars	Two (or more) children move (walking or running) in a disorderly manner and collide with each other as in a bumper car game
Chasing	The child runs after a companion to catch them
Dragging	The child grabs the companion by the legs, arms, and hands, dragging them a short distance
Fighting	This is a wrestling game, during which two (or more) children may roll, climb on top of each other, give bites, slaps, punches, and kicks
Fleeing	The child runs to avoid being caught by their chasing companion
Hide-and-Seek	The child hides from their companion's view, who starts searching for them until they find them
Hitting with object	The child uses an object (e.g., a toy) to beat any part of the companion's body
Jumping	The child moves their body upwards, lifting their feet off the ground and then returns downwards, touching their feet on the ground together with a companion. The child may also jump onto a part of the companion's body
Kicking	The child quickly bends and straightens their legs together with a companion while standing or sitting or rapidly extends a leg to hit the companion
Object play	Two or more children play together with a toy or an object used as a toy
Picking a fellow	The child lifts a companion in a ventral or lateral position, either staying in place or walking for a short distance
Pirouetting	The child spins around while standing or performs a somersault together with a companion
Pretend play	The pretend play can also be referred to as symbolic play (Piaget, 1964) or fantasy play (Smith, 2009). Within pretend play, three distinct types of symbolic transformations have been identified (Stone, 2007; Smith, 2009): i) object transformation—this involves utilizing an object as something different, for instance, using a banana as a pretend telephone; ii) role transformation—children engage in role-playing by imitating and adopting various roles, like pretending to be a doctor or a dancer; and iii) ideational transformation—the child constructs a fantasy using language, gestures, or mental images that are divorced from actual objects; for instance, a child might use gestures and language to enact a surgical procedure
Pulling	The child quickly moves a companion towards them by grabbing their legs, arms, hands, or another part of the companion's body
Pushing	The child moves a companion away by using hands or feet. This pattern also includes pushing a companion on a bike/tricycle/toy car
Recovering a thing	The child snatches an object from the companion's hands and then flees, being chased in turn
Retrieving	The child restrains the companion to prevent them from leaving
Running	Two (or more) children move rapidly in a parallel way
Sheltering	The child uses their arms, hands, or an object to shield themselves from the attack by the companion
Slapping	The child uses the palm of the opened hand to hit any part of the companion's body
Social painting	The child can draw or color together with a companion by using the same sheet
Tickling	The child uses their hands, feet, head, or mouth for rubbing a part of the companion's body
Turning around	The child circles around an object (e.g., table, chair, tree) together with a companion. The same behavior can also be performed by two or more subjects while holding hands in contact
Walking on	A child strolls on top of the companion while they are lying on the ground
Wriggling	A child rapidly moves their body to break free from the grip of the companion and release themselves

For both smiling and yawning, a facial expression from the trigger was considered as perceived if it fell within the visual range of the potential responder, that is the face orientation of the potential responder was: i) frontal to the trigger; ii) diagonal, requiring a 45° head rotation to reach the frontal vision of the trigger; iii) lateral, requiring a 90° head rotation to reach the frontal vision of the trigger (Norscia & Palagi, 2011).

**Fig. 1:**
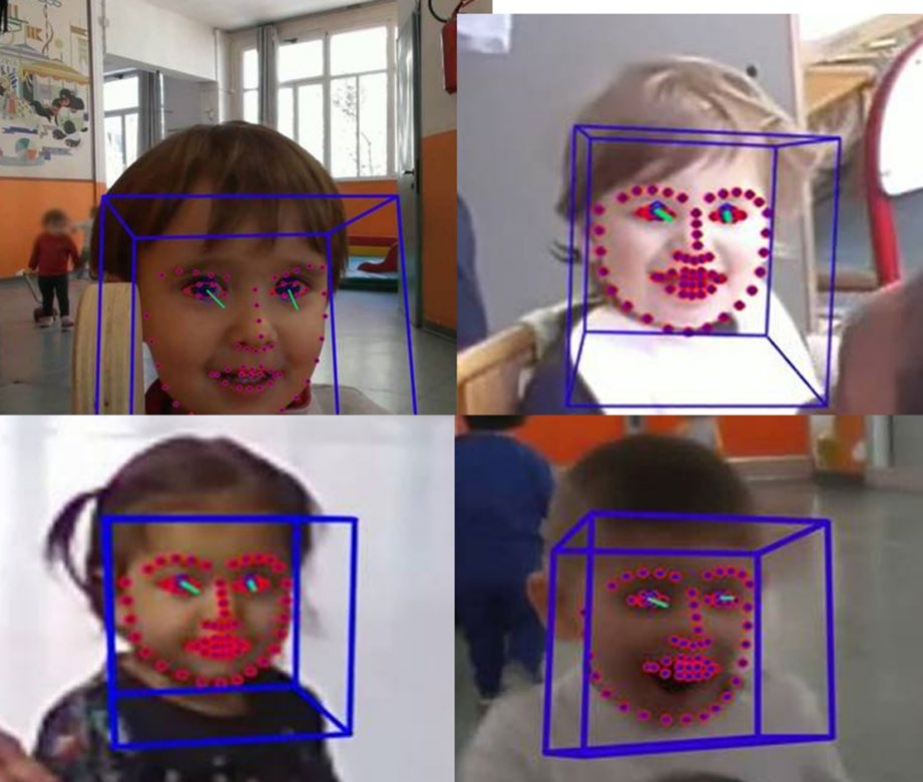
Smiles considered in this study, preliminarily identified via Openface/FACS. The image shows Openface elaboration. Top left: basic/simple smile (mouth closed/no eye constriction; involved AUs: AU12, lip corner puller, *zygomaticus major*; AU14, dimpler, *buccinator*; AU45, blink, *levator palpebrae*, *orbicularis oculi*, pars *palpebralis*); top right: play smile (mouth open, no eye constriction; involved AUs: A12 and A14; AU4, brow lowerer, *depressor glabellae*, *depressor supercilli*, *currugator*; A6, cheek raiser, *orbicularis oculi*, *pars orbitalis*; A25, lips part, *depressor labii*, *orbicularis oris*); bottom left: Duchenne smile (mouth closed/eye constriction; involved AUs: AU6, AU12, AU14); bottom right: duplay smile (mouth open/eye constriction; involved AUs: AU2, outer brow raiser, *frontalis*, *pars lateralis*; A6; A12; A25; A26, jaw drop; *masetter*; *temporal and internal pterygoid*)

To check for the presence of Rapid Smile Mimicry (RSM), we considered four types of smile: simple/basic smile (mouth closed/no eye constriction), play smile (mouth open/no eye constriction), Duchenne smile (mouth closed/eye constriction), and duplay smile (mouth open/eye constriction; Fogel et al., 2006; Messinger et al., 2008). The different types of smiles were first identified on 20% of the video-recorded and clearly visible facial expressions using—depending on the image quality—FACS (Facial Action Coding System; Ekman & Friesen, 1978) and freeware OpenFace 2.0 (Ambadar et al., 2009; Baltrušaitis et al., 2018). FACS identifies the contraction of 33 facial muscle AUs and allows the comparison of facial movements regardless of face morphology variation (Waller et al., 2020). OpenFace 2.0 is able to detect facial AU activation also when the face is non-frontal and/or in low illumination conditions (Amos et al., 2016). These tools were complementarily used to preliminarily check for mouth and eye opening/closing, and using faces as a starting recognition point in naturalistic videos. Figure 1 shows the four types of smiles with the Facial Action Units involved.

We considered the smiles emitted by a toddler (potential responder) within 1 s after they observed (sight condition) or did not observe (no sight condition) a previous smile (triggering expression) emitted by the playmate (trigger). A smile was considered as perceived when it fell within the visual field of the potential responder based on their head orientation (see above for definition). If the potential responder was initially looking away but then turned their face towards the trigger while it was still smiling, this case was considered as perceived. Any ambiguous or doubtful case was excluded from the analyses.

For Yawn Contagion (YC), we recorded the yawns emitted by a toddler within 3 min after they observed (sight condition) or did not observe (no sight condition) a previous yawn (triggering expression) by another classmate (the trigger). A yawning pattern was recognized as such when it involved jaws open in a wide gape, deep inhalation, eye closing or narrowing (open yawns; Norscia et al., 2021; Provine, 2012). Considering that yawn detection is not axially specific (meaning that yawns in orientations of 90°, 180°, and 270° are as potent as frontal yawns in releasing a yawning response; Norscia & Palagi, 2011; Provine, 1989a, 1989b, 1996), a yawn was classified as perceived if the potential responder, with eyes open, had the face of the trigger in their field of vision (see below for definition). A yawn was considered unperceived if the potential responder had their head turned 180° away from the trigger, or when there was an obstacle between the subjects that obscured the responder's view. Only one yawn in the dataset involved the use of vocal folds and was considered as perceived. Doubtful situations were excluded from the analyses. The 3-min span is the time window that is most used in literature (Gallo et al., 2021; Norscia & Palagi, 2011; Valente et al., 2023) and that reduces the likelihood of autocorrelation, maximum in the fourth minutes (autocorrelation occurs when a yawn emitted by an individual at t_0_ increases the probability that the same individual will repeat yawning at t_(0+X)_, where X is the increasing unit of time; Kapitány & Nielsen, 2017). To further reduce autocorrelation likelihood, in case of a yawning chain (i.e., several yawns emitted in a row by the same toddler during 3 min, with no other individual yawning), we considered as a response only the first yawn emitted after the last triggering yawn (Gallo et al., 2021). When more than one yawning response occurred from different subjects, the first responder would become a trigger.

The frequency of affiliation between toddlers was calculated as the number of affinitive interactions (as described in Table 1) normalized over the dyad observation time. Given the variety of countries of origin of the toddlers’ parents, we included the ‘geographic background’ in our analysis. Two toddlers were considered to have the same geographic background if at least one parent from each toddler shared the same country of origin.

### Statistics

The RFM presence analysis involved a total of 33 dyadic combinations, 3.61 ± 0.64 (Mean ± SE) whereas the YC presence analysis involved 195 dyadic combinations, 17.80 ± 0.74 (Mean ± SE).

Owing to the dataset, not large enough to allow the separation into different smile types, we conflated the four smile types into a single category ‘smile’. The first two Generalized Linear Mixed Models (GLMMs) were run to test for the presence of either Smile Facial Mimicry (RSM; GLMM_1a_; N_events_ = 119) or Yawn Contagion (YC; GLMM_2a_; N_events_ = 3474). The binomial target variable was the presence/absence of either smile (GLMM_1a_) or yawn (GLMM_2a_). In GLMM_1a_ we included as fixed factors, the perception condition (binomial, no sight/sight) and the class the toddlers belonged to (binomial, Lavender/Pebbles); in GLMM_2a_ we included same fixed factors plus distance (multinomial: contact/proximity/non-proximity, within 10 m/non-proximity more than 10 m).

Then, considering the cases where the triggering signal (smile or yawn) was perceived, we ran two GLMMs to verify what individual factors possibly influenced either RSM (GLMM_1b_; N_events_ = 78) or YC (GLMM_2b_; N_events_ = 149; for this analysis we considered only the cases where the stimulus came from a single trigger). We included the presence/absence of either RSM (GLMM_1b_) or YC (GLMM_2b_) as the binomial target variable. The fixed factors for either model were the sex of trigger and potential responder (binomial, male/female), trigger/potential responder age (numeric variable) and geographic background (binomial, same/different background). As a control, we ran two mirror models for either RSM (GLMM_1b_CONTROL_; N_events_ = 41) or YC (GLMM_2b_CONTROL_; N_events_ = 3318), with the same exact factors but considering the cases where the triggering signal was *not* perceived by the potential responder (that is the ‘response’, if any, was actually a spontaneous, untriggered emission of the facial expression).

Finally, considering—as above—the cases where the triggering signal (smile or yawn) was perceived, we ran two GLMMs to verify whether affiliation levels possibly influenced either RSM (GLMM_1c_; N_events_ = 78) or YC (GLMM_2c_; N_events_ = 149; for this analysis we considered only the cases where the stimulus came from a single trigger). We included the presence/absence of either RSM (GLMM_1c_) or YC (GLMM_2c_) as the binomial target variable and the affiliation level as fixed factor (numeric). As a control, we ran two mirror models for either RSM (GLMM_1c_CONTROL_; Nevents = 41) or YC (GLMM_2c_CONTROL_; N_events_ = 3318), with the same factor (affiliation level) but considering the cases where the triggering signal was not perceived by the potential responder.

In all these models, dyad identity was included as a random factor.

To compare the full (including all the considered fixed factors) and the null model (including only the random factors) (Forstmeier & Schielzeth, 2011), we applied the likelihood ratio test (Dobson & Barnett, 2018), making the analysis of variance with argument “Chisq” by using the function “*glmer*” of the package “*lme4*” (Bates et al., 2015) of the statistic program R (R Core Team, 2022; version 4.2.1). In case of significant difference between the full and the null model, we applied the R‐function “*drop1*” to extract the *p* values for each predictor included in the full model (Barr et al., 2013). Then, the effect size of each variable included in the full model was calculated via the package “*effect*”, with the function “*allEffects*”, and the confidence interval of each predictor using the function “*Confint*”. We applied the function *bobyqa* of the package *minqa* in case of non-convergence of the model (Powell, 2009).

## Results

### Rapid Smile Mimicry (RSM)

**Fig. 2:**
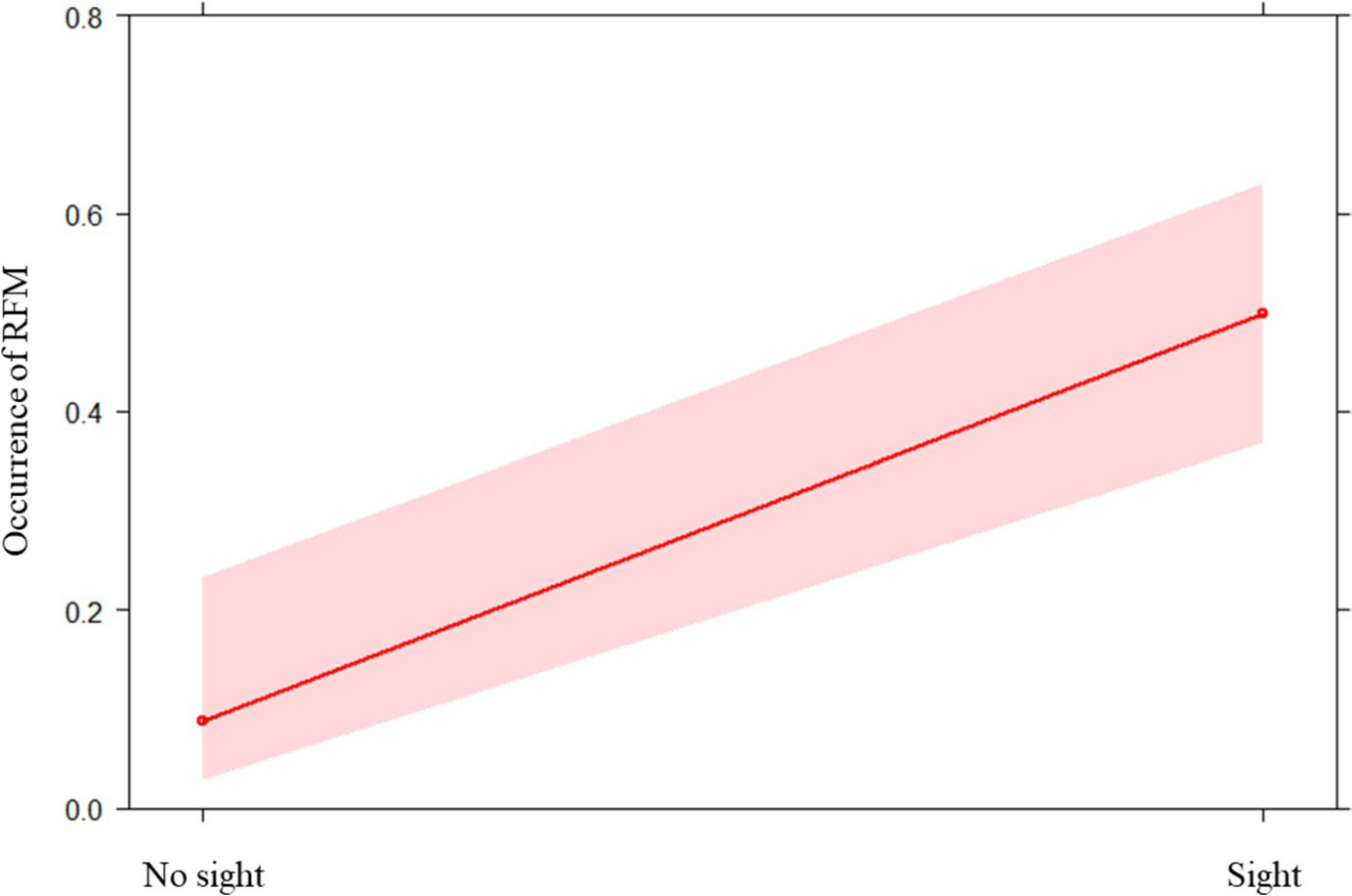
Effect plot about the occurrence of RFM, specifically RSM (Table 2). The occurrence (Y axis) was higher in the condition “Sight” than in the condition “No sight” (X axis). The confidence interval is represented by the band

**Fig. 3:**
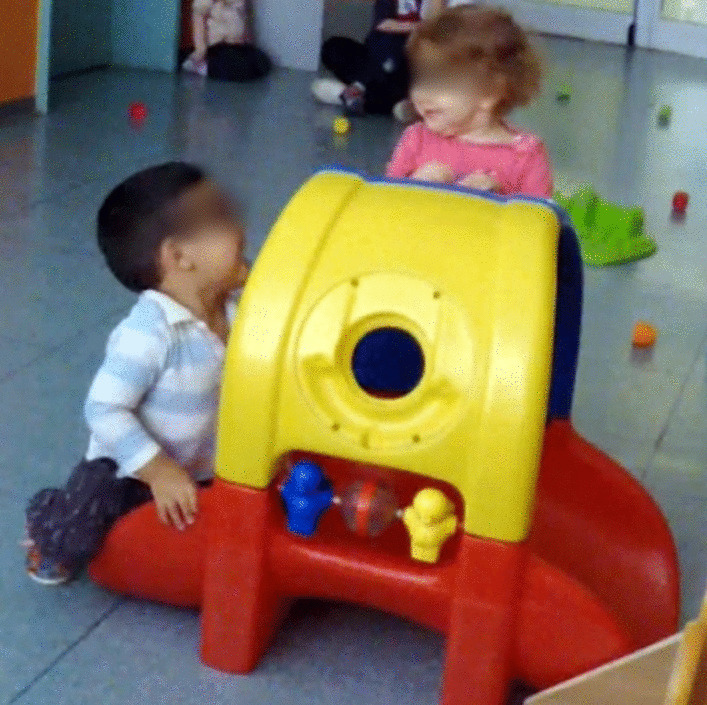
Example of smile mimicry

**Fig. 4:**
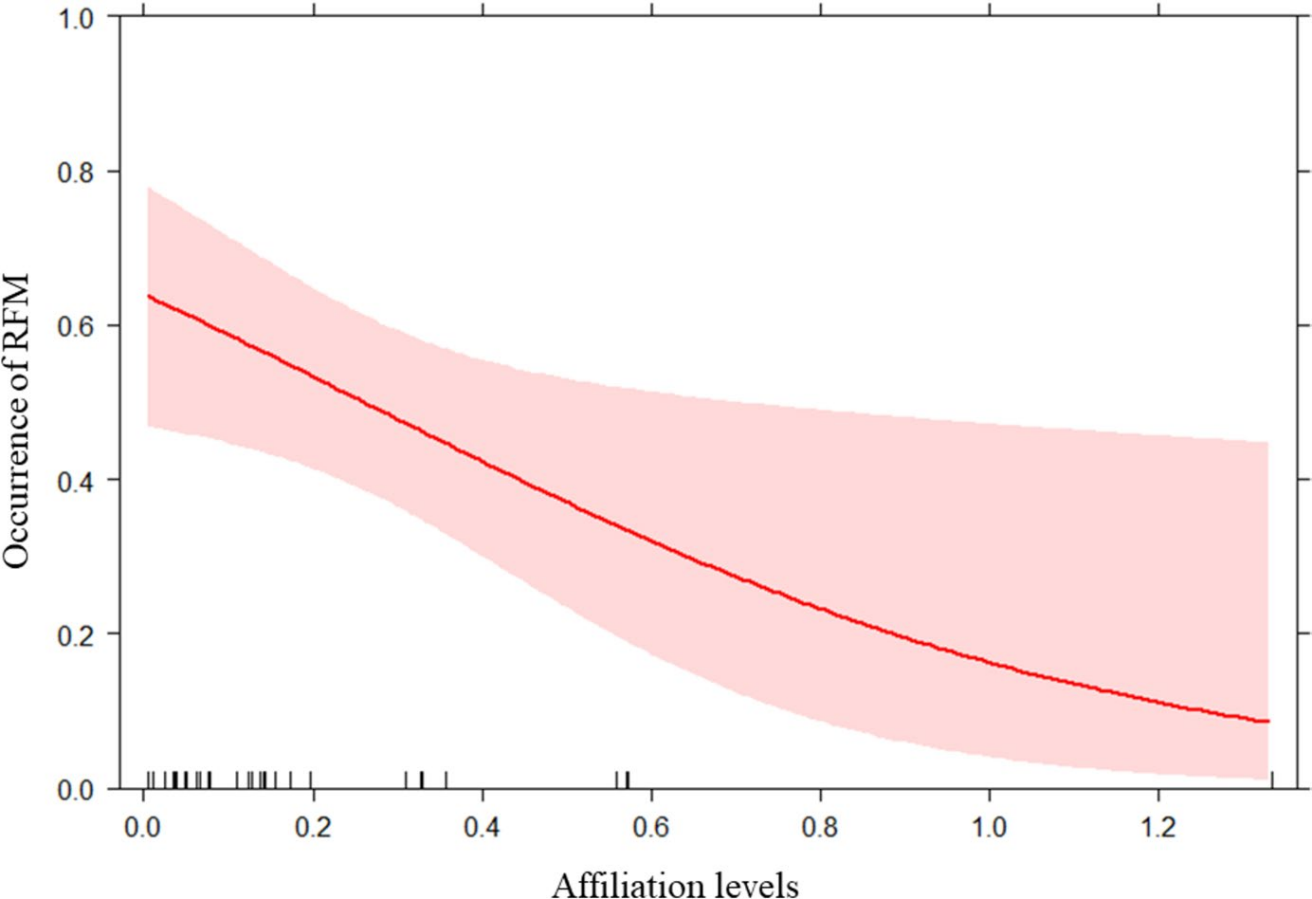
Effect plot about the effect of the Affiliation levels between trigger and responder on the occurrence of RFM, specifically RSM (Table 2). The occurrence (Y axis) increased when the Affiliation levels (X axis) decreased. The confidence interval is represented by the band. The rug plot at the bottom of the graph shows the location of the affiliation level values

We ran the GLMM_1a_ to check for the presence of RSM. We found a significant difference between the full and the null model (likelihood ratio test: x^2^ = 20.753; df = 2; *p* < 0.001). Hence, the fixed factor variable had a significant main effect on the dependent variable (*p* < 0.001; see Table 2). In particular, it was significantly more likely that a child performed a smile after observing a smile emitted by the playmate than when no previous smile was observed (Fig. 2 and Fig. 3). We found no significant difference between the full and the null model for GLMM_1b_ on individual modulating factors (age, sex, and parents’ country of origin; likelihood ratio test: x^2^ = 7.457; df = 5; *p* = 0.189). Hence, no variable had a significant effect. Regarding the GLMM_1c_ on social modulation, we found that the full model significantly differed from the null model (likelihood ratio test: x^2^ = 5.558; df = 1; *p* = 0.018), with a significant effect of the variable “affiliation levels” (*p* = 0.028; see Table 2). Specifically, we found that the response probability increased as the affiliation levels decreased (Fig. 4). Finally, as regards the two control models (GLMM_1b_CONTROL_ and GLMM_1c_CONTROL_) we found no significant difference between the full and the null model for both of them (GLMM_1b_CONTROL_: likelihood ratio test: x^2^ = 7.553; df = 5; *p* = 0.183; GLMM_1c_CONTROL_: likelihood ratio test: x^2^ = 0.208; df = 1; *p* = 0.648).

**Table 2:** Full results of: GLMM_1a_ on the presence of RSM (N_events_ = 119); GLMM_1b_ and GLMM_1c_ on the possible influence of individual and social factors on the presence of RSM (N_events_ = 78); and GLMM_1b_CONTROL_ and GLMM_1c_CONTROL_ as control models (N_events_ = 41)

Predictors	Estimates		SEM	CI_95_	Effect size	×^*2*^		p
GLMM_1a_	Full vs. null model: ×^2^ = 20.753; *df* = 2; *p* < **0.001**							
(Intercept)^a^	−2.195	0.796		−3.755; −0.635	a	a		a
Sight (yes)	2.344	0.666		1.039; 3.648	0.499	3.522		** < 0.001**
Classroom (Lavender)^b^	−0.182	0.647		−1.450; 1.087	0.301	−0.281		0.779
GLMM_1b_	Full vs. null model: ×^2^ = 7.457; *df* = 5; *p* = 0.189							
GLMM_1c_	Full vs. null model: ×^2^ = 5.558; *df* = 1; *p* = **0.018**							
(Intercept)^a^	0.577	0.357		−0.123; 1.277	a	a	a	
Affiliation level	−2.213	1.010		−4.194;−0.234	0.638	−2.191	**0.028**	
GLMM_1b_CONTROL_	Full vs. null model: ×^2^ = 7.553; *df* = 5; *p* = 0.183							
GLMM_1c_CONTROL_	Full vs. null model: ×^2^ = 0.208; *df* = 1; *p* = 0.648							

^a^ Not shown as not having a meaningful interpretation

^b^ These predictors were dummy-coded, with the reference category as follow: Sight = no; Classroom: “Pebbles”

### Yawn Contagion

**Fig. 5:**
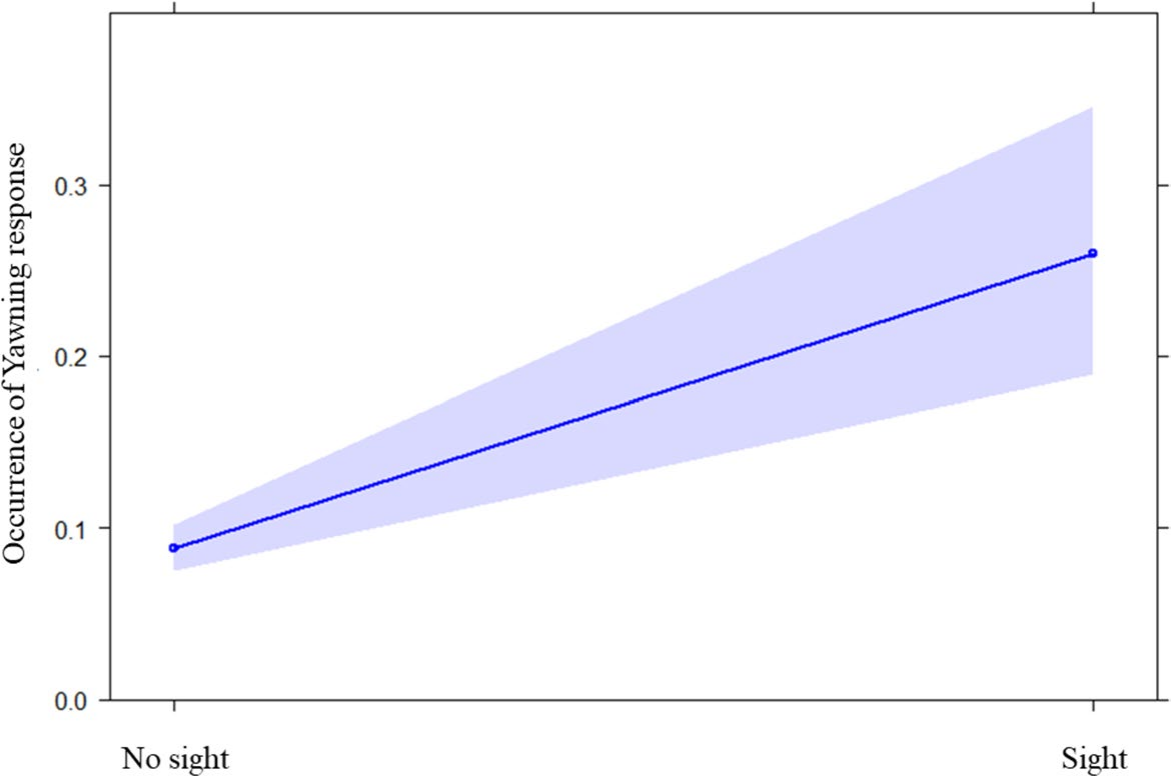
Effect plot about the occurrence of yawning response (Table 3). The occurrence of yawning response (Y axis) was higher in the condition “Sight” than in the condition “No sight” (X axis). The confidence interval is represented by the band

**Fig. 6:**
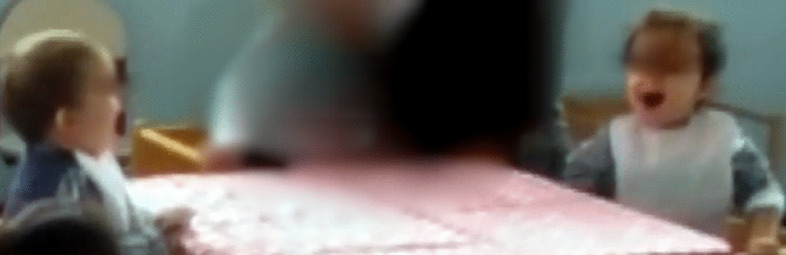
Example of yawn contagion

**Fig. 7:**
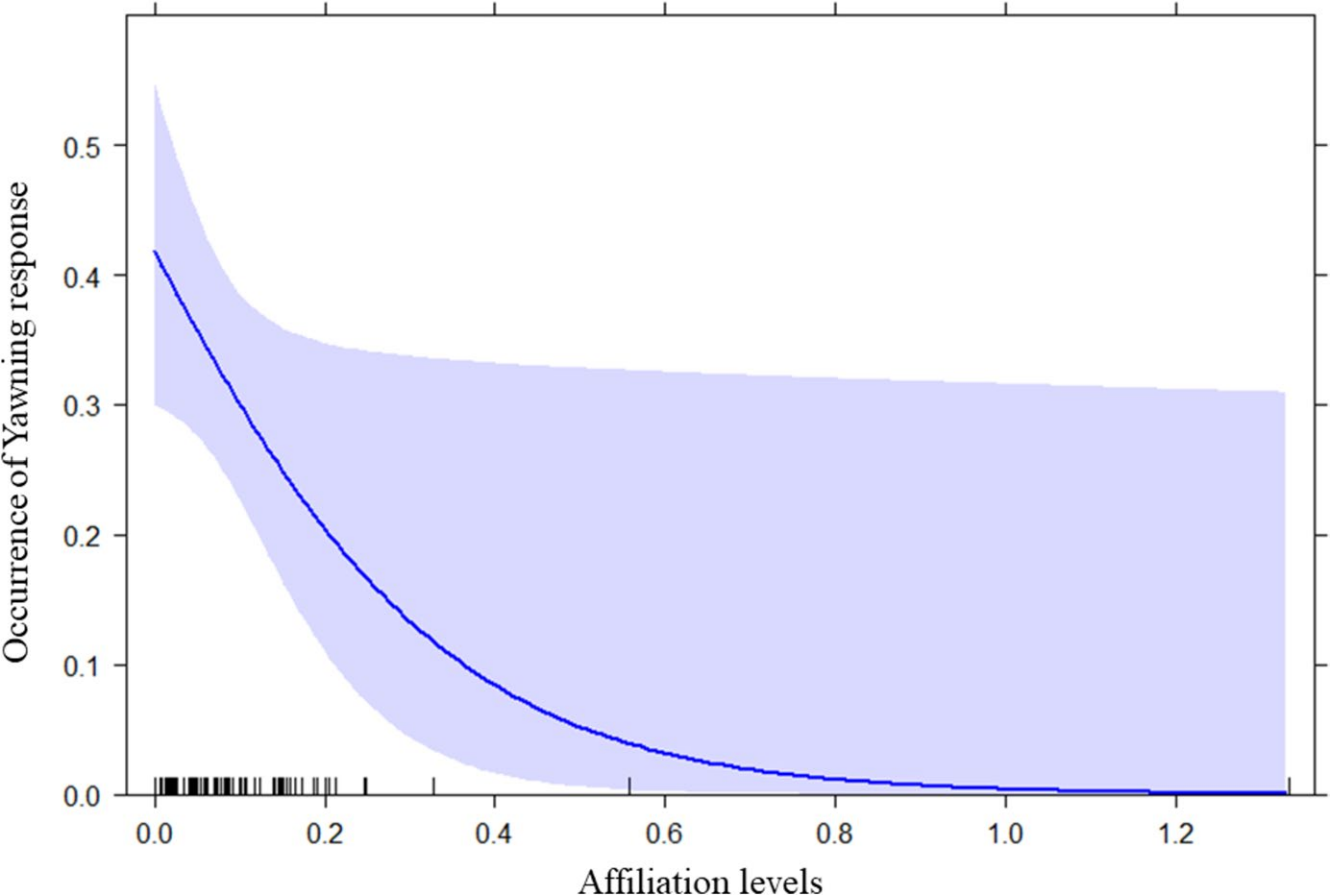
Effect plot about the effect of the Affiliation levels between trigger and responder on the occurrence of yawning response (Table 2). The occurrence of yawning response (Y axis) increased when the Affiliation levels (X axis) decreased. The confidence interval is represented by the band. The rug plot at the bottom of the graph shows the location of the affiliation level values

We found a significant difference between the full and the null model for GLMM_2a_ on presence/absence of yawn contagion (likelihood ratio test: x^2^ = 46.846; df = 5; *p* < 0.001). Hence, we moved on with a drop1 procedure. Only the perception variable had a significant main effect on the dependent variable (*p* < 0.001; see Table 3). In particular, it was significantly more likely that a toddler yawned after perceiving a previous yawn from another child than when no previous yawn was detected (Fig. 5 and Fig. 6). GLMM_2b_ on individual factors did not reveal any significant difference between the full and the null model (likelihood ratio test: x^2^ = 2.236; df = 5; *p* = 0.816). Therefore, none of the variables considered (age, sex, and parents’ country of origin) had an effect on yawn contagion. Instead, we found a significant difference between the full and the null model related to GLMM_2c_ on social modulation (likelihood ratio test: x^2^ = 8.986; df = 1; *p* = 0.003), with the occurrence of YC being highest as the levels of affiliation between toddlers were lowest (*p* = 0.046; see Table 3 and Fig. 7). Regarding the GLMM_2b_CONTROL_ on individual factors, we found a significant difference between the full and the null model (likelihood ratio test: x^2^ = 21.534; df = 5; *p* = 0.001) with a significant effect of the variable “responder sex” (*p* < 0.001; see Table 3) which revealed that males yawned more than females. Finally, as regards the GLMM_2c_CONTROL_ on potential effect of affiliation level, we found no significant difference between the full and the null model (likelihood ratio test: x^2^ = 0.916; df = 1; *p* = 0.338).

**Table 3:** Full results of: GLMM_2a_ on the presence of yawn contagion (N_events_ = 3474); GLMM_2b_ and GLMM_2c_ on the possible influence of individual and social factors on the presence of yawn contagion (N_events_ = 149); and GLMM_2b_CONTROL_ and GLMM_2c_CONTROL_ as control models (N_events_ = 3318)

Predictors	Estimates	SEM	CI_95_	Effect size		x^*2*^	p
GLMM_2a_	Full vs. null model: ×^2^ = 46.846; *df* = 5; *p* < **0.001**						
(Intercept)^a^	−2.637	1.120	−4.831; −0.441		a	a	a
Sight (yes)	1.295	0.211	0.881; 1.708		0.260	6.137	** < 0.001**
Classroom (Pebbles)^b^	0.130	0.156	−0.176; 0.437		0.101	0.834	0.404
Distance (Proximity)^b^	0.482	1.123	−1.720; 2.682		0.113	0.429	0.668
Distance (Within 10 m)^b^	0.217	1.118	−1.975; 2.408		0.089	0.194	0.846
Distance (More than 10 m)^b^	0.046	1.577	−3.044; 3.136		0.076	0.029	0.977
GLMM_2b_	Full vs. null model: x^2^ = 2.236; *df* = 5; *p* = 0.816						
GLMM_2c_	Full vs. null model: x^2^ = 8.985; *df* = 1; *p* = **0.003**						
(Intercept)^a^	−0.330	0.264	−0.847; 0.186	a		a	a
Affiliation levels	−5.133	2.578	−10.186; −0.081	0.418		−1.991	**0.046**
GLMM_2b_CONTROLLO_	Full vs. null model: x^2^ = 21.534; *df* = 5; *p* < 0.001						
(Intercept)^a^	−1.836	0.273	−2.371; −1.302	a		a	a
Trigger sex (Female)^b^	−0.035	0.141	−0.312; 0.241	0.085		−0.251	0.802
Responder sex (Female)^b^	−0.620	0.144	−0.901; −0.338	0.065		−4.315	** < 0.001**
Trigger age	0.010	0.011	−0.011; 0.032	0.101		0.921	0.357
Responder age	−0.019	0.011	−0.040; 0.002	0.111		−1.795	0.073
Geographic background (Different)^b^	0.060	0.146	−0.225; 0.346	0.090		0.414	0.679
GLMM_2c_CONTROLLO_	Full vs. null model: x^2^ = 0.016; *df* = 1; *p* = 0.338						

^a^ Not shown as not having a meaningful interpretation

^b^ These predictors were dummy-coded, with the reference category as follow: Sight: “no”; Classroom: “Pebbles”; Distance: “Contact”; Trigger sex: “Male”; Responder sex: “Male”; Geographic background: “Same”

## Discussion

Our results indicate that motor replication can be present in toddlers for both Rapid Smile Mimicry (RSM; Prediction 1a supported) and Yawn Contagion (YC; Prediction 2a supported). Effect sizes (Table 2 and 3) are not large but correspond to odds ratios (1.30–1.65) that are practically significant. The presence of RSM and YC in young toddlers is consistent with previous reports indicating that facial mimicry in humans appears in the first months of age (Ferrari et al., 2013; Isomura & Nakano, 2016). The emergence of motor replication in pre-verbal toddlers aligns with the observation that rapid facial mimicry and yawn contagion are rooted in human biology and anchored to the non-verbal domain, as they are also found in non-human mammals, including primates and particularly hominids (e.g. Anderson et al., 2020; Davila-Ross et al., 2008; Demuru & Palagi, 2012; Norscia et al., 2022; Palagi et al., 2019; Palagi et al., 2020; van Berlo et al., 2020; but see Palagi et al., 2019). Facial expressions can convey emotional cues and the ability to gain emotional information from others’ bodily and facial movements is primordial to survival (Heck et al., 2018). Indeed, emotion detection (for negative and positive emotions) involves a neural network that includes evolutionarily conserved brain areas such as the amygdaloid complex, in humans, non-human primates and other animals (Ferretti & Papaleo, 2019; Pabba, 2013). Motor resonance and the possibly related emotional sharing (a foundation of empathy) are drivers of prosocial behaviors in humans and other animals (Decety et al., 2016). Indeed, children that are better able to spot and share the expressions of their peers can be more prosocially responsive to their peers (Denham et al., 2003). In humans and other hominids, coordination of behaviors and internal states allows social interactions to successfully continue at the dyadic level and the achievement of collective goals at the group level (Nowak et al., 2017; Palagi et al., 2020; Parr et al., 2005). Focusing more specifically on yawn contagion, its presence also below two years indicates that this type of motor resonance emerges earlier in human development than previously described. Cordoni et al. (2021) pointed out that the naturalistic setting, and exposure to spontaneous, real yawns (rather video or faked stimuli) from peers belonging to the same group (rather than adults and/or unfamiliar subjects) allows the early detection of motor resonance. Such conditions may indeed increase signal effectiveness, reduce response inhibition and/or enhance the motoric response.

As concerns the modulating factor of rapid facial mimicry and yawn contagion, for neither phenomenon we found an effect of sex (as expected; Prediction 1c and 2c, for RSM and YC respectively, supported), age (contrary to expectations; Prediction 1b and 1c for RSM and YC respectively, not supported), and parents’ country of origin (the predictions diverged for RSM and YC, with an effect expected only for RSM; Prediction 1d on RSM not supported; Prediction 2d on YC supported). Regarding age, it is possible that the short age span of our toddlers—with age increasing by the month—has dampened the effect of age-related variations. It is also possible that in the early ontogenetic stages covered by this study, such variation has not yet emerged. Indeed, important changes in replication modulation abilities may emerge in toddlers at later stages and, for example for yawn contagion, a conspicuous increase probably starts occurring after 5 years of age (Anderson & Meno, 2003; Cordoni et al., 2021; Helt et al., 2010; Jones, 2009).

Regarding the geographical origin, the lack of an appreciable effect on both RSM and YC may be because the toddlers were born in the same country (although some of the parents had immigrated from abroad), they knew each other since at least 4–5 months, and belonged to the same class, hence to the same social group. Although an ethnic bias in facial recognition may exist and decrease with age, there is evidence that overall visual attention may not differ as a function of ethnicity, that happy faces are better identified across ethnic groups and that humans from different ethnic background can overcome such bias when they live together for a period of time (Kawakami et al., 2014; Michel et al., 2006; Segal et al., 2019). Regarding sex, the early stage of developments—far from gonadal maturation and related morphophysiological changes—may account for the lack of sex differences, which are instead observed later in life for facial mimicry (e.g., Dimberg & Lundquist, 1990) and yawn contagion (e.g., Chan & Tseng, 2017) at least in certain human cohorts. This is also consistent with the lack of sex effect on yawn contagion in children from 2.5 to 5.5 years old (Cordoni et al., 2021). It is interesting to notice, however, that in the control model on the individual factors modulating yawning (to be considered in this case as spontaneous as untriggered by previous yawns from others), the yawner sex had a significant effect, with males yawning more than females. Similarly, Cordoni et al. (2021) found that male children yawned more than female children under naturalistic conditions, possibly due to androgens, which are known to increase yawning in mammals (Cordoni et al., 2021; Graves & Wallen, 2006; Homgren et al., 1980; Melis et al., 1994; Rodriguez-Sierra et al., 1981) and can already have an effect in perinatal phases (Alexander, 2014; Vigil et al., 2016). The increased yawn levels in males may also be linked to stress-induced aggression and higher cortisol levels (Cordoni et al., 2021; Thompson, 2014), as male infants can be more easily aroused by stressors under certain conditions (Richardson et al., 2010). Further studies are needed to investigate this, as sex effects on spontaneous yawning in infants may vary with external stimuli (e.g., Menin et al., 2022 found increased yawning in infant females). It is essential to replicate this study across various toddler cohorts to see if the observed lack of RSM and YC modulation in relation to individual factors holds true at the population level for the age span considered here, or if variations may emerge based on age, sex, and geographic origin composition.

Finally, the affiliation level (a proxy for social bond) had an effect on both RSM and YC. Effect sizes (Table 2 and 3) are good and correspond to odds ratios that are practically significant (1.51–1.97). Our result is in line with the observation that the ability to differently respond to others’ emotional expressions depending on the level of attachment (i.e., familiarity) appears early in the development of infant (Walker-Andrews et al., 2011), along with the ability to distinguish emotional facial expressions emerges (5–7 months of age; Cruz et al., 2023; Flom & Bahrick, 2007). However, the most intriguing result is that the observed effect of affiliation levels was not in the expected direction (Prediction 1e and 2e for RSM and YC respectively, not supported). While in certain cohorts of human adults both RFM and YC can increase when the social bond is tighter (e.g., Fischer et al., 2012; Häfner & Ijzerman, 2011; Norscia & Palagi, 2011), in our toddler cohort we observed an opposite effect. Specifically, RSM and YC were highest between toddlers sharing least affiliation. The social asymmetry in the levels of RSM and YC across dyads may indicate that they do not merely involve motoric resonance and that internal states may be also involved, depending on the experience that is shared with them (as predicted by the Perception–Action Model, de Waal & Preston, 2017). When the relation between motor resonance and social bond is positive (the former increases as the latter increase), it has been hypothesized that motoric resonance may be coupled with a basic form of empathy, as the positive association mirrors the so-called ‘empathic trend’ (Palagi et al., 2020; de Waal & Preston, 2017; but see: Massen & Gallup, 2017). Instead, when the relation between motor replication phenomena and social bond is not positive, but as in our case negative, the link between motoric resonance and emotional contagion must be interpreted within a broader framework. Even though accounts are scarce at the moment, there is evidence that in non-human hominids motor replication can have a negative relation with affiliation levels. As a matter of fact, this trend has been observed for example in young gorillas for RFM (Bresciani et al., 2022) and in bonobos for yawn contagion (De Vittoris et al., 2024). The variable association between motor replication and affiliation levels may be rooted in human biological history. The amygdala, an evolutionarily conserved brain area, in humans encodes episodic memory and subjective evaluation of emotional faces rather than just visual elements (Dolcos et al., 2004; Wang et al., 2014). This is an adaptive feature, as natural selection favors responses that are most suitable to individuals in the environment where they interact. Indeed, environment modulates such responses (Bijlsma & Loeschcke, 2005). Facial expression phenotypes and functions are connected to socio-ecological context (social intelligence hypothesis; Schmidt & Cohn, 2001). In humans, contextual factors may influence how social factors modulate facial expression replication (Seibt et al., 2015), and context variety and social information complexity can obscure emotional cues from facial movements (Barrett et al., 2019). Context can also determine the emotional nuance of expressions and the corresponding response, as valence is not always entirely evident (Kret & Akyuz, 2022). Yawning in humans may be associated for example with testosterone (anger), cortisol (distress) but also with neutral behavioral transitions related to the circadian rhythm (e.g., Cordoni et al., 2021; Thompson, 2014; Zilli et al., 2007). Smile is more widely associated with happiness but in both adult humans and children it may be expressed out of frustration or in social exclusion situations (incongruent affect; Mateo Santana & Grabell, 2023; Svetieva et al., 2019). Young children are still in the process of building their competence in the finely tuned detection of emotions in others’ facial expressions, which is important for effective socio-emotional communication (Dehnam, 2018). The young children under study included infants and early toddlers with still scarce experience in emotionally interacting with others in a group social setting. Both inexperience and complex social situations may have contributed to shaping their response to the facial expressions of group mates in relation to affiliation. Motor replication, coupled with emotional state replication, may function in reducing the prediction error over others’ behavior, thus leading to appropriate decision making (Kret & Akyuz, 2022). The decision-making process, in humans, also involves subcortical brain areas (Prochazkova & Kret, 2017). The reduction of the prediction error may lead to at least to two opposite outcomes (and of course a range of possibilities in-between). On one extreme, such reduction may be pivotal to the continuation of social interaction via coordination, as the prediction error is probably highest between less familiar individuals. This may help develop social bonds with less known individuals (De Vittoris et al., 2024). Depending on the circumstances, however, the error reduction may also serve to interrupt, rather than facilitate, an interaction (Kret & Akyuz, 2022; Diana & Kret, 2025). In humans, for example, mimicry can lead to lower levels of trust (Diana et al., 2023) and yawn can mark behavioral transitions, which involve the interruption of one activity to commence another (e.g., resting to moving, sleep to wake; Gallup, 2022; Zannella et al., 2015). Based on the above elements, it is possible to point out that the social asymmetry of motor replication in relation to affiliation observed in toddlers suggests that internal states may be also involved in such replication. The type of emotional connection that is established via mirror social releasers can depend—especially in toddlers—on the stage of development of individual competence and various contextual variables, such as group composition and situational aspects. This kind of modulation emerges early in ontogeny and is probably evolutionary ancient, for the reasons explained above.

This study can lay the groundwork for deeper investigation in various complementary directions. In order to do that, because young toddlers socially play at lower frequencies than older ones, larger datasets are necessary. For example, future work may consider the possible laterality of emotional facial expressions (e.g. Mandal and Ambady, 2004) and whether mimicry is (or not) axially specific, as these aspects may also modulate the response. The combined effect of RSM and YC on affiliation levels may also be considered, as long with the effect of the mimicry of different types of smiles, here conflated into a single category. A point of reflection concerns the fact that in a naturalistic context it was not possible to measure eye contact, which is a factor known to potentially influence facial mimicry, particularly in the context of smile mimicry (Mauersberger et al., 2022). However, this study has the merit of considering mimicry in its naturally occurring social context, and in this respect, our results can add to the results obtained by using eye-tracking techniques. Future studies should explore the effect that mimicry has on play sessions (e.g., duration, patterns used) to highlight possible repercussions on social bonding. Moreover, the inclusion of other human cohorts (at early and later developmental stages) and the adoption of comparative approach with other primates (especially hominids) would be welcome in future investigation, to delve further into the ontogenetic and evolutionary basis of facial mimicry. Finally, in this study, we specifically focused on smile mimicry and contagious yawning, but other potential dynamics between dyads are likely to exist, such as mimicry of different facial expressions or behaviors. This aspect is worth considering in future investigations, possibly on a larger dataset.

## Data Availability

The data associated with this research are available at this link: https://github.com/inorscia/yawn_smile_toddlers_2024.git.
